# Extension of interval between adjacent pulse delivery cycles to deal with myocardial ischemia by intravascular lithotripsy: case report

**DOI:** 10.1186/s13019-024-02782-z

**Published:** 2024-05-04

**Authors:** He Lv, Xinyu Li, Zengduoji Ren, Zhilu Qin, Chunying Fu, Qiang Fu

**Affiliations:** Department of Cardiology, The People’s Hospital of Liaoning Province, Wenyi Road, Shenhe, Shenyang, Liaoning Province 110016 China

**Keywords:** Intravascular ultrasound, Intravascular lithotripsy, ST-segment depression, Shockwave balloon

## Abstract

**Background:**

Intravascular lithotripsy (IVL) represents a novel approach in the management of coronary calcification. This technique employs acoustic pressure waves, generated by a shockwave balloon, to effectively fracture both superficial and deep calcification in situ. The efficacy and safety of IVL have been convincingly demonstrated through the Disrupt CAD I-IV studies. While IVL is associated with the occurrence of atrial and ventricular arrhythmias, there is no evidence to indicate it causes myocardial ischemia.

**Case Description:**

A 71-year-old man was admitted presenting with chest pain. His previous coronary angiography revealed stenosis and calcification in the left anterior descending branch. An attempt to predilate the lesion using two Lacrosse non-slip element balloons was unsuccessful. Ventricular premature beats and transient ST-segment depression were captured during the utilization of IVL. The operator gradually extended the pulse emission interval across two consecutive cycles to mitigate myocardial ischemia. Notably, when the interval reached 30s, the patient had no chest pain or ST-segment changes. Subsequent images of intravascular ultrasound confirmed calcification ruptures. Therapeutic intervention included the placement of a stent and the application of a drug-coated balloon in the left anterior descending branch. A telephonic follow-up six months later indicated the patient had no discomfort.

**Conclusions:**

This case underscores the effectiveness of gradually extending the pulse emission interval as a strategic complement to the clinical application of IVL. In certain clinical scenarios, it may become imperative to suspend the pulse delivery to improve myocardial blood supply.

**Supplementary Information:**

The online version contains supplementary material available at 10.1186/s13019-024-02782-z.

## Introduction

Intravascular lithotripsy (IVL), heralded as “a disruptive technology”, is utilized to treat coronary calcification. The mechanism of action involves the generation of an electric spark by IVL, which causes the mixed liquid in the balloon to vaporize, forming bubbles. These bubbles expand and collapse rapidly, exerting disruptive forces on the calcified plaques [1]. The Disrupt CAD series studies [2–5], encompassing a total of 626 patients, demonstrated no reflow or slow flow. However, in the Disrupt CAD III study, there were 2 severe dissections, 1 perforation and 1 acute occlusion. While IVL is known to potentially induce atrial or ventricular arrhythmias, there is no literature about myocardial ischemia caused by the technology [4]. This case focuses on the causes and management strategies of transient ST-segment depression triggered by IVL.

This manuscript is written following case reporting checklist.

## Case presentation

**Fig. 1:**
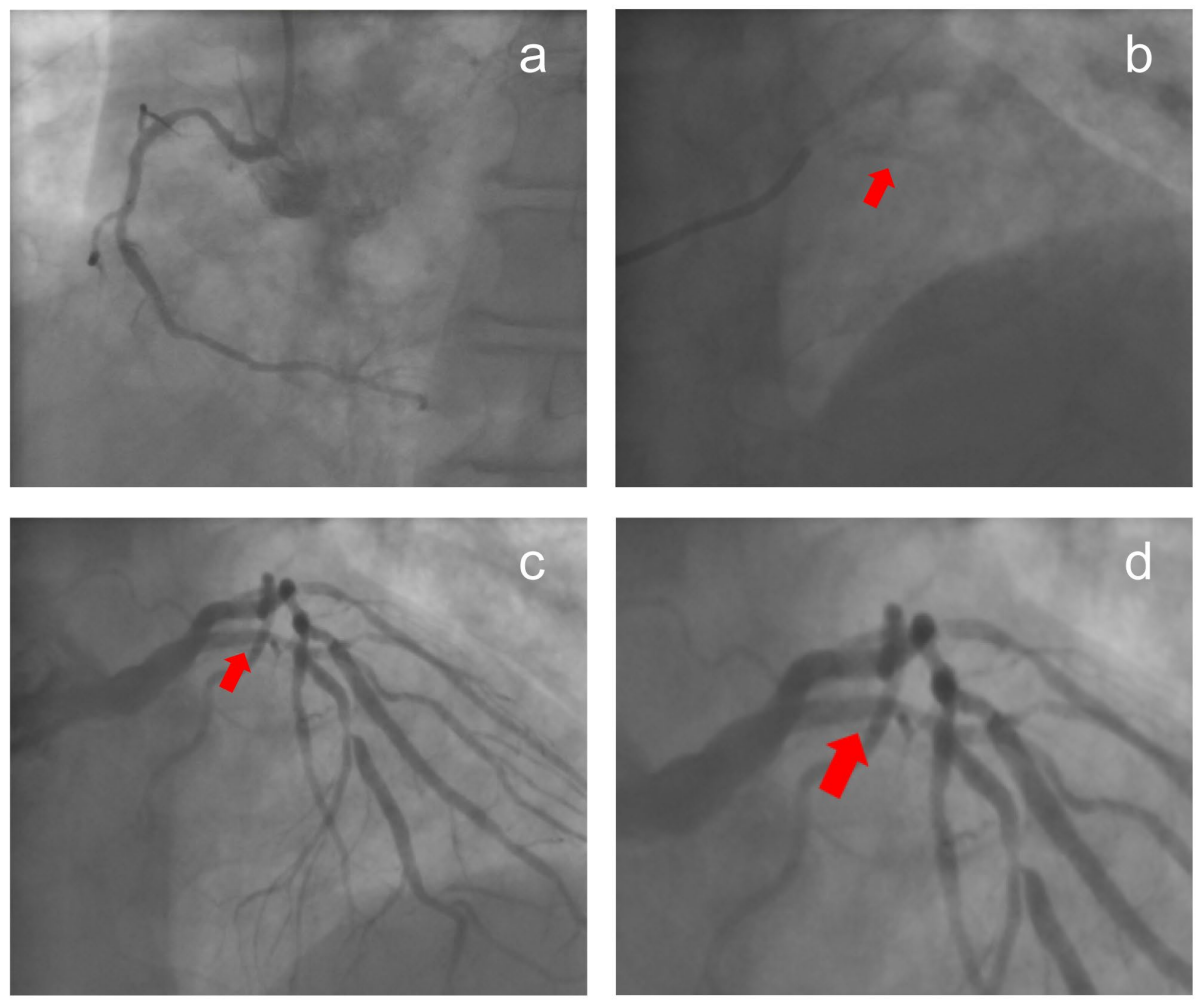
Coronary angiography images. **a** is an image of right coronary artery. **b** is the calcification in the left anterior descending branch without contrast media. **c** is the calcification in the left anterior descending branch with contrast media. **d** is the amplification of local lesions from c. The red arrows are calcifications in the four images

**Fig. 2:**
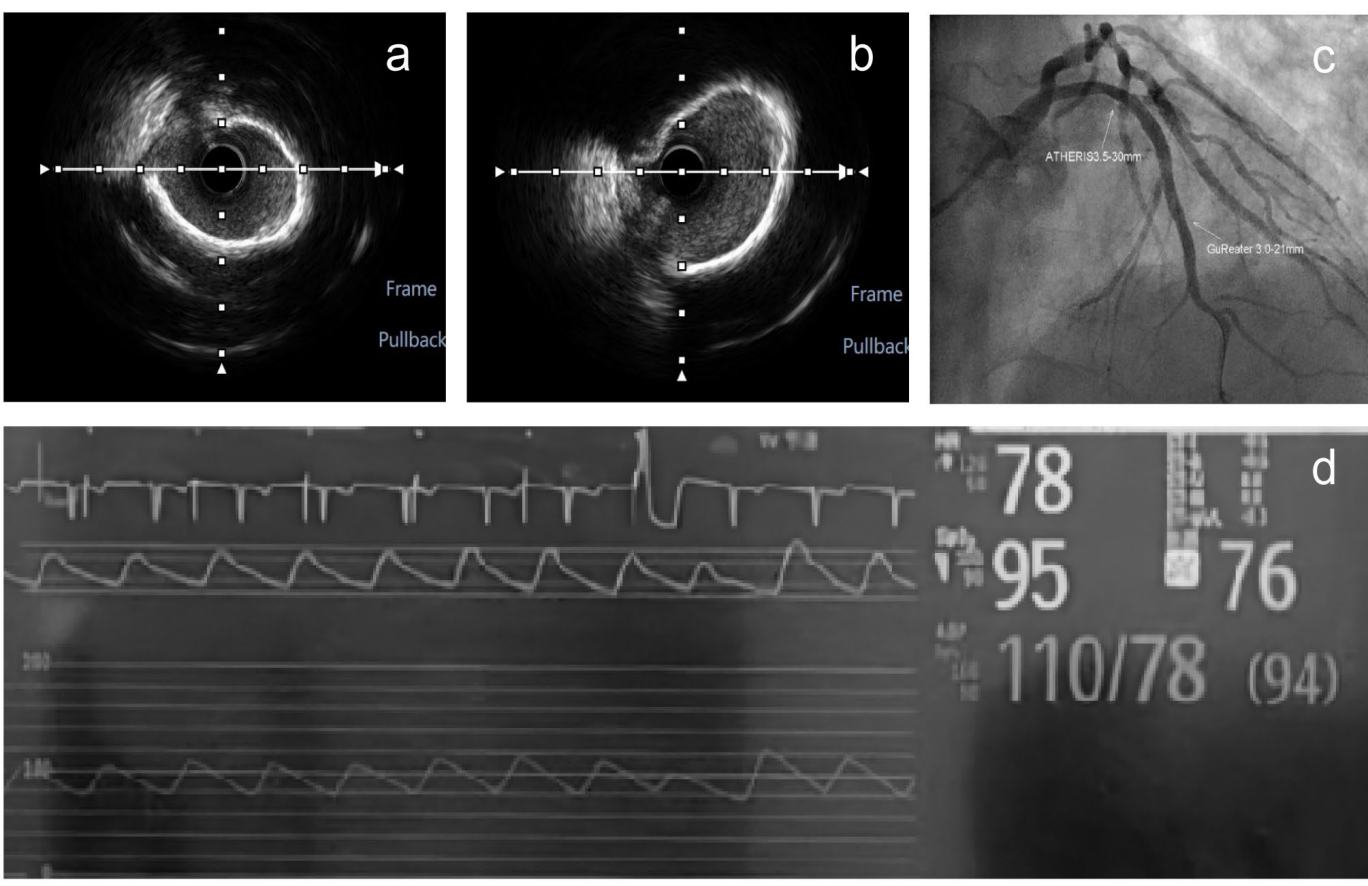
Auxiliary examinations related to intravascular lithotripsy. **a** is an image of intravascular ultrasound before intravascular lithotripsy. **b** is an image of intravascular ultrasound after intravascular lithotripsy. **c** marks the location of the stent (3.0 mm*21 mm) and drug balloon (3.5 mm*30 mm). **d** shows a ventricular capture

**Fig. 3:**
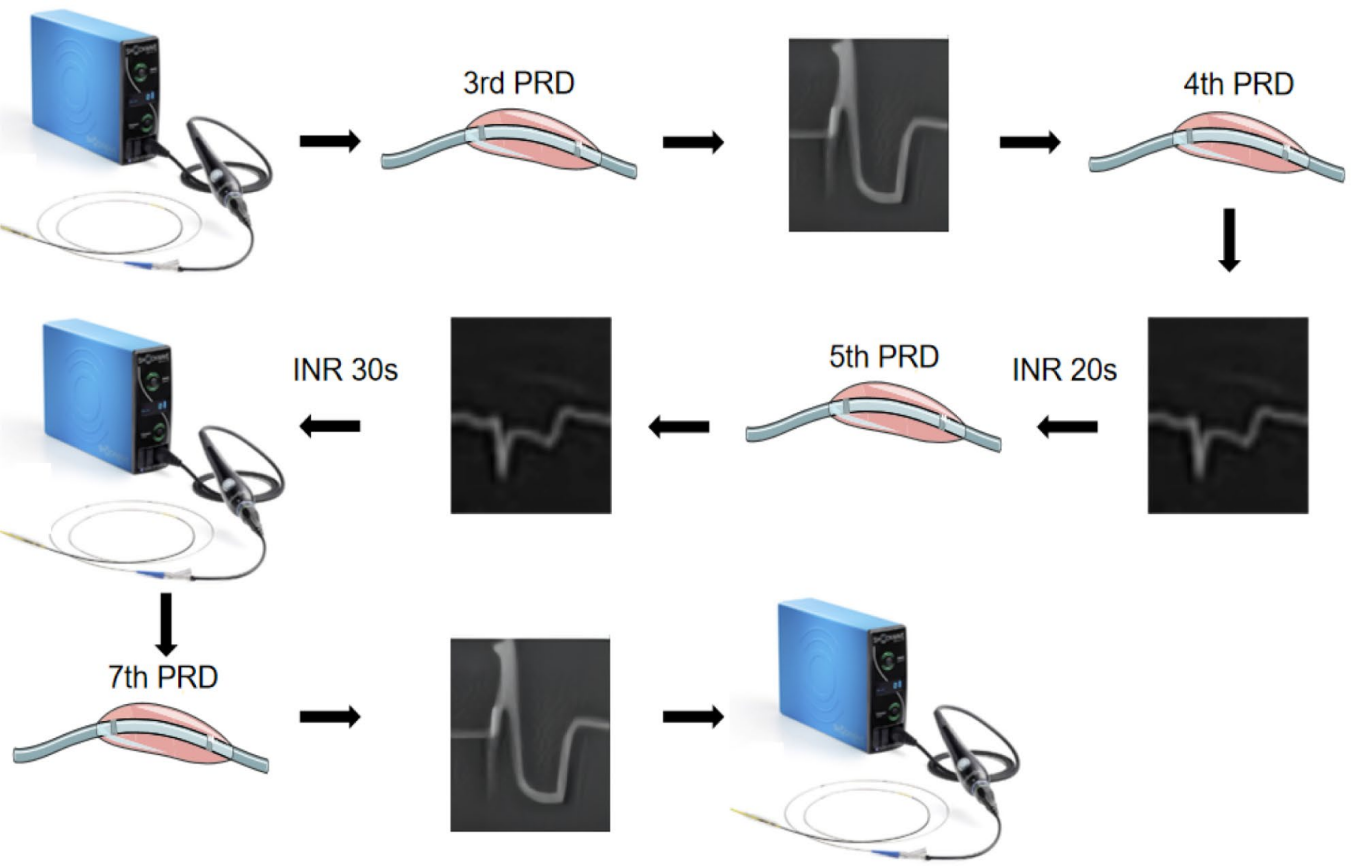
Management process of intravascular lithotripsy-induced myocardial ischemia. A 4.0 mm*12 mm shockwave balloon was progressed with 8 cycles. In the third and seventh period, IVL induced ventricular premature beats, and the phenomenon was not being addressed. Chest pain and transient ST-segment depression were developed in the fourth period by IVL. After balloon deflation, there was nothing. For the symptom and ST-segment depression, the operator extended the interval between the pulse delivery of adjacent cycles to 20s. The patient still had chest pain and transient ST-segment depression in the fifth period. For the conditions, the operator extended the interval to 30s and the symptom and ST-segment changes disappeared. IVL, intravascular lithotripsy; INR, interval; PRD, period

A 71-year-old male with smoking history was admitted due to recurrent angina. His previous coronary angiography showed stenosis with calcification in the left anterior descending branch (LAD) and thin right coronary artery (Fig. 1). Physical examination indicated no other discomfort. His electrocardiogram demonstrated inverted T waves in limb lead. The post-admission test results were normal. Based on the clinical findings, the patient was diagnosed with unstable angina. The therapeutic regimen included antiplatelet agents, coronary vasodilators and lipid-lowering agents. One day later, intravascular ultrasound (IVUS) confirmed the diffuse lesion from the LAD ostium to the middle section (minimum lumen area: 2.24mm^2^, plaque load: 80% and a grade-IV calcification in the Fig. 2). Two Lacrosse non-slip element balloons (3.0/3.5 mm*10 mm) failed to predilate the lesion. A 4.0 mm*12 mm shockwave balloon was progressed with 8 cycles. IVL not only induced ventricular premature beats, but also caused chest pain symptoms and transient ST-segment depression on the electrocardiogram (Additional file 1). After balloon deflation, there was nothing. The operator extended the interval between the pulse delivery of adjacent cycles to 20s. The patient still had chest pain and transient ST-segment depression. The duration of a single ST-segment depression was between 11 and 13s. For the above conditions, the operator extended the interval to 30s and the chest pain symptoms or ST-segment changes disappeared (Fig. 3). Subsequently, calcification ruptures were seen in the IVUS (Fig. 2a-b). After implantation of a stent in mid-LAD, a drug balloon was performed in the proximal of LAD (Fig. 2c), and final angiography and IVUS displayed good results. The results of the postoperative test were normal. Electrocardiogram showed no significant change compared to preoperation after operation. A telephone follow-up six months later indicated the patient had no discomfort.

## Discussion

In this case, ventricular premature beats and transient myocardial ischemia were captured during the operation. According to the literature, the equipment of IVL can produce low and rapidly decayed energy (8–10µJ) and activate stretching activation channels in the cardiac conduction system. Furthermore, myocardium can be depolarized, leading to arrhythmia [6]. In this case, IVL induced isolated ventricular premature beats, which did not necessitate medical intervention.

It has been reported that ventricular premature beats can exacerbate the progression of atherosclerosis and enhance plaque instability by affecting hemodynamics. In this case, the presence of ventricular premature beats may affect the blood flow supply of coronary arteries to some extent [7]. Repeated interruption of blood flow leads to ischemia further, which causes clinical symptoms and ST-segment changes. The operator did not interrupt the pulse delivery, but continued to lengthen the interval for restoring distal blood supply. Fortunately, when the interval was extended to 30s, the patient had no chest pain or ST-segment changes. Follow-up after discharge also indicated a good prognosis of the patient.

## Conclusions

The way about gradual extension of the pulse emission interval complements the clinical application of IVL. Depending on the specific clinical scenarios, it may be necessary to improve myocardial blood supply by suspending pulse delivery. And this method of lengthening the interval needs to be further validated safety and efficacy in a large number of clinical trials.

### Electronic supplementary material

Below is the link to the electronic supplementary material.

**Additional file 1**: Electrocardiogram monitor companied with ventricular caputures and transient ST-segment depression

## Data Availability

Not applicable.

## Declarations

### Footnote

Patient Perspective: The operation is a great success. I have had no chest pain since the stent was implanted, and I will be checking back for coronary angiography.

## Abbreviations

IVL: intravascular lithotripsy
IVUS: intravascular ultrasound
LAD: left anterior descending branch

## References

[1] Lee MS, Kereiakes DJ, Shlofmitz RA (2023). Intravascular lithotripsy for calcified Left Main Artery Disease. J Soc Cardiovasc Angiogr Interv.

[2] Brinton TJ, Ali ZA, Hill JM (2019). Feasibility of Shockwave Coronary intravascular lithotripsy for the treatment of calcified coronary stenoses. Circulation.

[3] Ali ZA, Nef H, Escaned J et al. Safety and effectiveness of coronary intravascular lithotripsy for treatment of severely calcified Coronary stenoses: the disrupt CAD II study. Circ Cardiovasc Interv. 2019;12(10):e008434. 10.1161/CIRCINTERVENTIONS.119.008434. 10.1161/CIRCINTERVENTIONS.119.008434 31553205

[4] Hill JM, Kereiakes DJ, Shlofmitz RA (2020). Intravascular lithotripsy for treatment of severely calcified coronary artery disease. J Am Coll Cardiol.

[5] Saito S, Yamazaki S, Takahashi A (2021). Intravascular lithotripsy for Vessel Preparation in severely calcified coronary arteries prior to Stent Placement - Primary outcomes from the Japanese disrupt CAD IV study. Circ J.

[6] Kereiakes DJ, Virmani R, Hokama JY (2021). Principles of intravascular lithotripsy for calcific plaque modification. JACC Cardiovasc Interv.

[7] Germanova O, Shchukin Y, Germanov V (2022). Extrasystolic arrhythmia: is it an additional risk factor of atherosclerosis?. Minerva Cardiol Angiol.

